# Traumatic right diaphragmatic rupture with hepatothorax in Ghana: two rare cases

**DOI:** 10.11604/pamj.2019.33.256.17061

**Published:** 2019-07-26

**Authors:** Isaac Okyere, Perditer Okyere, Paul Sedem Komla Glover

**Affiliations:** 1 Cardiovascular and Thoracic Surgery Unit, Department of Surgery, Kwame Nkrumah University of Science and Technology, Komfo Anokye Teaching Hospital, Kumasi, Ghana; 2 The Renal Unit, Department of Internal Medicine, Kwame Nkrumah University of Science and Technology, Komfo Anokye Teaching Hospital, Kumasi, Ghana; 3 Department of Emergency Medicine, Komfo Anokye Teaching Hospital, Kumasi, Ghana

**Keywords:** Traumatic right diaphragmatic injury, hepatothorax, thoracotomy, blunt and penetrating chest injury

## Abstract

A rare case series of traumatic right diaphragmatic rupture with hepatothorax in Ghana is reported. The first case involved a middle-aged man who sustained a penetrating chest injury following an unprovoked attack by a wild bull. The second case was a young woman who sustained a blunt chest injury after being knocked down by a moving vehicle whiles crossing the road. Both presented with ruptured right diaphgramatic rupture and had to undergo repair through thoracotomy after stabilization and the two had been well one year after surgery without any complications or sequelae.

## Introduction

Traumatic injuries of the diaphragm remain an entity of difficult diagnosis despite having been recognised early in the history of surgery. Traumatic diaphragmatic injury may result from a penetrating injury or blunt thoracoabdominal injury and results in communication between the pleural and peritoneal cavities. The injury can occur either on the right or on the left hemidiaphragm, however the left diaphragm is more commonly involved, as its weakest point is located on the left posterolateral aspect of the pleuroperitoneal membrane. On the other hand, the right diaphragm is able to withstand greater intra-abdominal pressure gradients due to the shock absorptive protection by the liver, therefore traumatic right diaphragmatic injury is a rare entity. The organs that most commonly herniate into the thorax include the stomach, spleen, colon, small bowel and liver. Their presentation can be immediate or delayed and they are often in combination with other more severe injuries especially the right side injuries. The diagnosis is missed among those admissions in up to two thirds of cases. Various modalities of imaging studies such as X-ray, sonography, multislice computerized tomography scan, magnetic resonance, thoracoscopy, laparoscopy and fluoroscopy are available for diagnosing diaphragmatic injury but the diagnostic image of choice is multislice computerized tomography. Delay in presentation can lead to complications and increased mortality.

## Patient and observation

### Case 1

**Figure 1:**
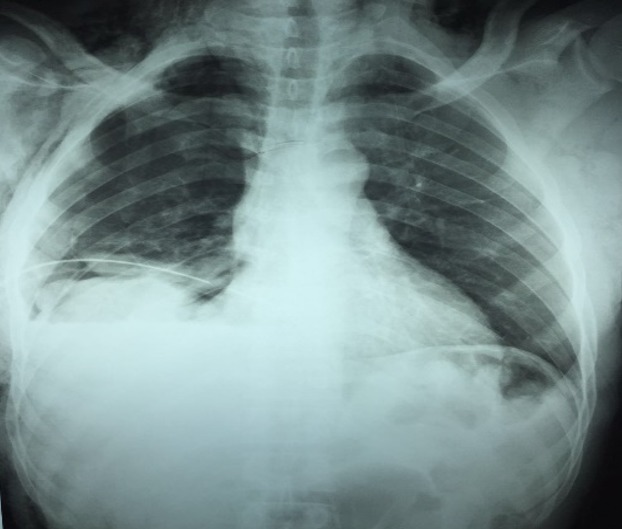
Preoperative erect chest x-ray of case 1 showing an irregularly shaped homogenous opacity in the right lower zone, loss of the right hemidiaphragmatic contour, a chest tube and minimal subcutaneous emphysema

**Figure 2:**
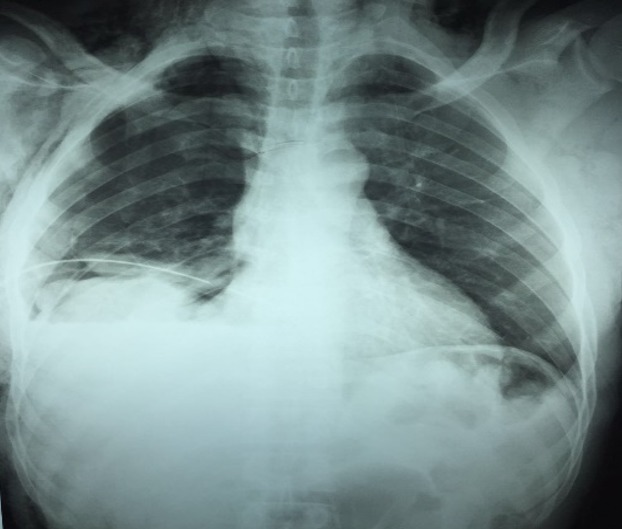
Preoperative chest CT scan of case 1 showing a complete rupture of right hemidiaphragm with herniated liver with capsular laceration

**History**: a 44 year old male cattle farm help and an alcohol abuser but a non-smoker with no significant past medical or surgical, chest or abdominal trauma history, was referred to the Accident and Emergency Centre of the Komfo Anokye Teaching Hospital from a peripheral hospital, two days after having been attacked by a bull in the chest. Patient was swang about in air three times while still attached to the bull, dropped onto the floor and stomped on in the right hemithorax and right upper abdomen before the bull was chased off by fellow workers. He presented to a peripheral hospital soon after the incident with a complaint of chest pain and was given analgesics, antibiotics and tetanus prophylaxis after evaluation and had his chest wounds dressed. Imaging could not be done at the facility. He is said to have momentarily improved, but continued to have right chest and abdominal pains and was thus referred to the Komfo Anokye Teaching Hospital on the third day of injury. On arrival he was conscious and alert and well oriented with SPO2 of 96% on room air, respiratory rate of 26 breaths/minute, heart rate of 124 beats/minute with a blood pressure of 137/57mmHg. His random blood sugar (RBS) was 8.6mmol/l, haemoglobin level of 10.9g/dl and had a temperature of 360°C. Systemic review was significant for shortness of breath, chest pain, cough (non-productive) and abdominal pain. He was talking clearly and there were no concerns for the airway and cervical spine. Chest expansion was limited on the right hemithorax with extensive subcutaneous emphysema from the jugular notch to the umbilicus and reduced breaths sound. There was a 2x2cm deep wound on the right anterolateral chest wall which was not communicating with the right pleural space. He had right upper abdominal guarding and tenderness. There were no significant pelvic and musculoskeletal findings except a clean 5x2cm laceration on the posterolateral aspect of the left thigh. He had warm peripheries with normal capillary refill time. Focused assessment with sonography for trauma (FAST) was negative for haemopericardium and haemoperitoneum. The patient was started on intranasal cannula oxygen therapy at 4L/min making SPO2 of 99%. Based on the clinical signs of right pleural collection as noted above, a size 32FG right chest tube was passed into the fourth intercostal space, midpoint between the anterior axillary line and the midaxillary line draining gush of air. The wounds were debrided cleaned and sutured under local infiltration. After twelve hours on admission, his haemoglobin had dropped to 7.9g/dl from the admission haemoglobin level of 10.9g/dl requiring two (2) units of packed red blood cell transfusion. His renal function was normal. Abdominopelvic ultrasound scan did not show much. Chest radiography and chest CT scan confirmed a right diaphragmatic injury with herniated liver as shown in Figure 1 and Figure 2. Patient was prepared for exploratory right thoracotomy.

**Figure 3:**
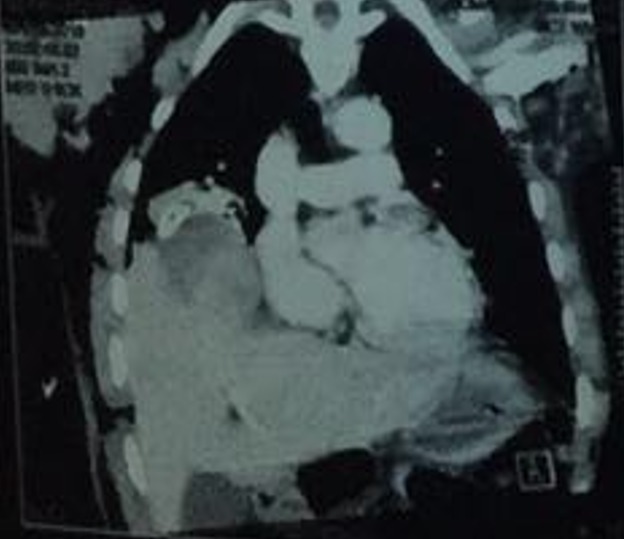
Intraoperative pictures of case 1 showing ruptured diaphragm with herniated liver and the repaired diaphragm

**Operation**: under general anaesthesia with a size 37FG double-lumen endotracheal tube, the patient was placed in a left lateral decubitus position and a standard right posterolateral thoracotomy was done entering the 7^th^ intercostal space or pleural bed. There was a 15cm transverse laceration of the right hemidiaphragm with herniation of the liver into the thoracic cavity, 280mls of hemothorax, fracture of the right 7^th^ and 8^th^ ribs and a collapsed and contused right lower lobe lung with intact pericardium, shown in Figure 3. The diaphgramatic rupture was repaired in a simple interrupted fashion, in a single layer using nylon 1 after reducing the liver into the abdomen. The chest was then irrigated copiously with warm normal saline. A size 32 FG chest tube was passed and the chest closed up in layers. He was extubated on table and had an uneventful postoperative recovery. The chest tube was removed on postoperative day 3 and he was discharged on postoperative day 10.

**Postoperative course**: he had a regular outpatient reviews and has had no complications or sequelae so far after one year.

### Case 2

**History**: 33-year-old lady was rushed from a peripheral hospital 13 hours after being knocked down by a moving vehicle while crossing a road to the accident and emergency centre of the Komfo Anokye Teaching Hospital. She was referred because of profuse scalp bleeding and transient loss of consciousness after the accident and persistent chest pains. She had been given antibiotics, analgesia and antitetanus prophylaxis. On admission, she was semiconscious with a GCS Score of 1115, M 5 V4 E2, moving with assistance, BP; 102/65 mmHg, heart rate of 108bpm, RR; 20 cycles/minute and temperature of 350°C.

**Figure 4:**
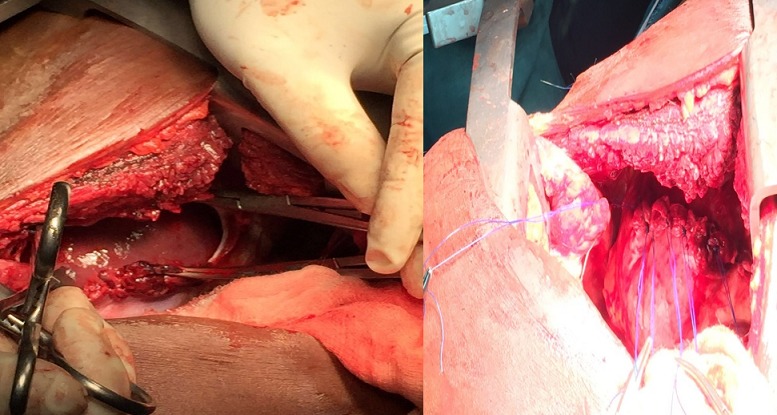
Preoperative chest x-ray of case 2 showing right chest opacity with loss of right diaphragmatic contour and contralateral shift of the medisternum

There was positive chest compression tenderness with reduced air entry on the right hemithorax but minimal abdominal signs. There was a wide abrasion injury on the right temporal region of the scalp, a laceration on the right parietal region and avulsion injury of the helix of the right ear with exposure of the cartilage. EFAST was positive for a right pleural effusion but negative for haemoperitoneum and haemopericardium. Patient was quickly stabilized with i.v fluids, antibiotics and the avulsed ear and the right parietal laceration were repaired by the maxillofacial surgeons. An urgent head CT scan showed no fractures or intracranial collections, but a chest radiograph showed massive opacity of the right hemithorax as shown in the Figure 4 and the loss of the right hemidiaphgramatic contour. Laboratory investigation showed haemoglobin level of 12.5g/dl, white cell count (WBC) of 20.83 and normal renal function. A size 28 FG right chest tube was passed quickly and blood was group-match against 4 units of packed red cells. The patient had become fully conscious after 18 hours of resuscitation and so she was wheeled to theatre for exploratory thoracotomy for a summary impression of mild head injury, avulsion injury of the right ear and right diaphragmatic rupture.

**Figure 5:**
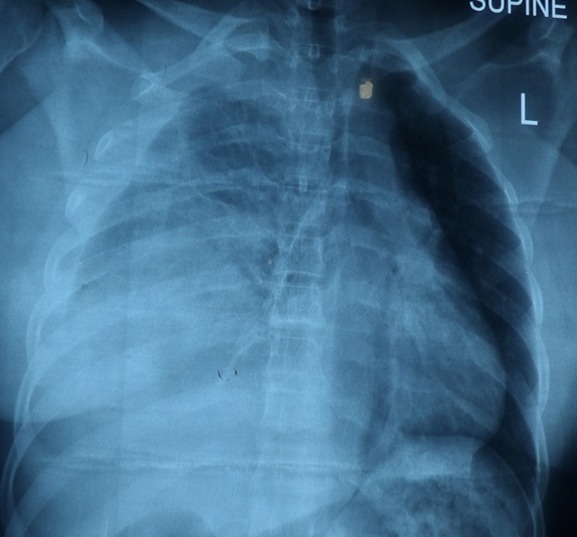
Intraoperative pictures of case 2 showing ruptured right diaphgram with herniated liver adherent to the edges and the postrepair

**Figure 6:**
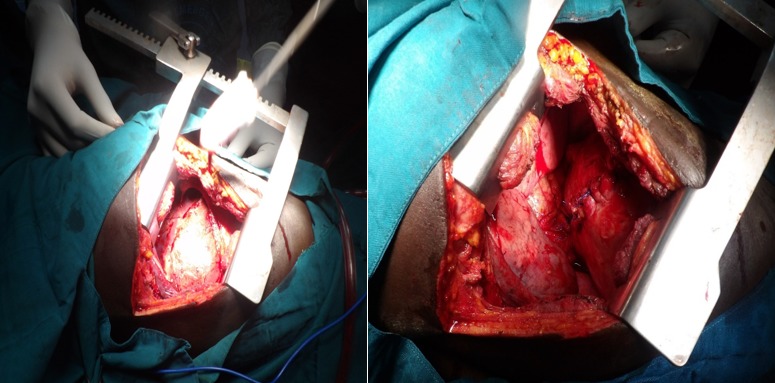
Postoperative erect chest x-ray of case 2 showing adequate bilateral lung expansion

**Operation**: under general anaesthesia with a size 37FG double lumen intubation, and in left lateral decubitus position, a standard right posterolateral thoracotomy was done entering the 7^th^ intercostal space. The findings included a complete transverse rupture of the right hemidiaphragm with fibrinous adhesions between the edges of the ruptured diaphragm and the herniated liver and 300mls of serosanguinous pleural effusion as shown in Figure 5. The adherent herniated liver was carefully freed from the diaphgramatic edges and reduced into the abdomen. The diaphragm was then approximated in a simple interrupted fashion in a single layer using Nylon 1. The chest was then irrigated with warm normal saline and a size 28 FG chest tube was inserted. Patient was extubated on table, chest tube removed on 4^th^ postoperative day and discharged on the 7^th^ day. She developed small right pleural effusion one week after discharge and superficial thoracotomy wound infection which were managed conservatively with antibiotics and she has been well one year after surgery with adequate bilateral lung expansion as shown in Figure 6.

## Discussion

Diaphragmatic ruptures can occur with both blunt and penetrating trauma which can be associated with intrathoracic herniation of abdominal viscera [1]. Following blunt thoracoabdominal trauma, diaphragmatic rupture is reported in 0.8-3.6% of patients [2]. Right-sided diaphragmatic rupture occurs in approximately 5-20% of all diaphragmatic injuries [2]. Left diaphgramatic ruptures are more common than the right because the right hemidiaphragm is mechanically stronger than the left and is also partially protected by the energy-absorbing liver. It therefore requires a greater force for injury [3, 4]. The mechanisms of rupture include a sudden increase in intra-abdominal pressure throughout the abdomen with the relatively weak unprotected left diaphragm tearing from the force or avulsion of the diaphragm from the chest wall, rib fracture fragments directly penetrating the diaphragm and direct injuries from impalement, stab or gunshot wounds [3, 4]. The physiopathological consequences of ruptured diaphragm affect mainly the cardiopulmonary system due to reduced function of the diaphragm, lung compression, mediastinal shift leading to impaired venous return to the heart and subsequently low cardiac output. Thoracic signs include decreased breath sounds, fractured ribs, flail chest, and signs of haemothorax or pneumothorax. Auscultation of bowel sounds in the chest is pathognomonic, especially in left-diaphgramatic ruptures due to thoracic intestinal herniation [1]. Abdominal signs include abdominal swelling, guarding, tenderness and absence of bowel sounds depending on the extent of injury. Occasionally physical examination can be relatively normal [5]. If diagnosis is delayed to months or years after the injury, symptoms are generally less severe, and are due to the space occupying lesion from the thoracic intestinal herniation (dyspnoea, orthopnoea, respiratory distress), and partial or complete obstruction of herniated abdominal contents (nausea, vomiting, abdominal, and chest pain). Our first patient sustained a penetrating injury to the chest from the horn of the bull leading to the rupture whereas the second patient sustained the injury form a blunt chest injury from the knocked down, however both presented with more chest signs than abdominal signs. The preoperative diagnosis of traumatic diaphragmatic rupture is difficult especially from penetrating injury [2, 3]. This was the situation with our first patient who was diagnosed on the third day of injury. Therefore, Sellke *et al*. have recommended exploratory laparotomy in patients with penetrating wounds inferior to the fourth intercostal space anteriorly (the nipple line in males), sixth interspace laterally, or eighth interspace posteriorly because of the contour and insertions of the diaphragm [4].

Chest X-ray is the first line of imaging study but the diagnostic imaging of choice is CT scan. Other imaging studies may include Ultrasound, MRI (magnetic resonance imaging), laparoscopy and thoracoscopy depending on the available of facilities and the haemodynamic state of the patient [1]. The diagnosis in the first patient was facilitated by the use of chest x-ray, abdominal ultrasound and finally confirmed by thoracoabdominal CT scan as shown in Figure 3 whereas only chest x-ray was used in the second patient as shown in Figure 4. Chest x-ray has a relatively low sensitivity for diagnosing diaphragmatic ruptures with only about 17-40% of the patients having suggestive signs [1]. The chest x-ray signs include abdominal gas patterns in the chest, nasogastric tube in the chest, blurring and elevation of the hemidiaphragm, mediastinal shift and compression atelectasis of the lower lung lobe [3]. There may also be pleural effusion (haemothorax), obliteration of the costophrenic angle and diaphragmatic contour distortion. These signs were shown in the chest x-ray of the two patients as shown in Figure 1 and Figure 4. The published diagnostic CT sensitivity and specificity of right hemidiaphragm rupture is 50-90% and 90-100% respectively [1-3]. MRI offers identical information to that of helical CT but with better direct coronal and sagittal images, however, restricted patient access, cost and unsuitability for emergency patients make MRI less preferable [1].

Immediate surgical management of acute traumatic diaphragmatic rupture via an abdominal approach is the current recommendation [3]. This facilitates the identification and management of associated intra-abdominal injuries, found in 30-70% of patients [3]. However, a thoracotomy approach should be used for all right diaphragmatic ruptures, regardless of the timing following initial injury [4], as was done for our patients, especially in the presence of minimal abdominal signs as also was found in both patient. Moreover there some absolute indications for a thoracotomy approach in diaphragmatic ruptures and this include diaphragmatic injuries involving the thoracic aorta, thoracoabdominal impalement injury, pericardiodiaphragmatic ruptures and severe contamination of the thoracic cavity. Repair of delayed diaphragmatic ruptures are also approached through thoracotomy [6]. Although this approach provides excellent exposure to divide the adhesions between the trapped viscera and lung parenchyma, a transabdominal approach may be preferable for a delayed left hemidiaphragm rupture in which segments of small or large bowel may have to be resected and anastomosed. Occasionally, a combination of laparotomy and thoracotomy may be preferred. The repair of the rupture is usually done with non-absorbable sutures and this can be done in either a continuous or interrupted fashion in a single or double layer. Nylon 1 repair in a simple interrupted fashion in a single layer was used for both patients. Synthetic grafts such as Dacron, Gore-Tex or Mesh may be needed if the laceration is large and the edges are rugged or there is extensive loss of tissue [1, 7]. The mortality associated with the right traumatic diaphragmatic rupture within the first 24hours is reported to be 0-31 % [1, 7].

## Conclusion

Traumatic right diaphragmatic rupture is an extremely rare entity, given the protection afforded by the liver and therefore the diagnosis of right diaphragmatic rupture requires a high index of suspicion. Although, chest X-ray is the most common initial imaging tool, CT scan is the preferred imaging modality. Immediate surgical repair via a thoracotomy approach to repair the diaphragm after reduction of the herniated abdominal contents constitutes the recommended acute surgical management.

## Competing interests

The authors declare no competing interests.
